# The N-Terminal GYPSY Motif Is Required for Pilin-Specific Sortase SrtC1 Functionality in *Lactobacillus rhamnosus* Strain GG

**DOI:** 10.1371/journal.pone.0153373

**Published:** 2016-04-12

**Authors:** François P. Douillard, Pia Rasinkangas, Arnab Bhattacharjee, Airi Palva, Willem M. de Vos

**Affiliations:** 1 Department of Veterinary Biosciences, University of Helsinki, Helsinki, Finland; 2 Research Programs Unit Immunobiology, Department of Bacteriology and Immunology, University of Helsinki, Helsinki, Finland; 3 Laboratory of Microbiology, Wageningen University, Wageningen, The Netherlands; University of Kansas Medical Center, UNITED STATES

## Abstract

Predominantly identified in pathogenic Gram-positive bacteria, sortase-dependent pili are also found in commensal species, such as the probiotic-marketed strain *Lactobacillus rhamnosus* strain GG. Pili are typically associated with host colonization, immune signalling and biofilm formation. Comparative analysis of the N-terminal domains of pilin-specific sortases from various piliated Gram-positive bacteria identified a conserved motif, called GYPSY, within the signal sequence. We investigated the function and role of the GYPSY residues by directed mutagenesis in homologous (rod-shaped) and heterologous (coccoid-shaped) expression systems for pilus formation. Substitutions of some of the GYPSY residues, and more specifically the proline residue, were found to have a direct impact on the degree of piliation of *Lb*. *rhamnosus* GG. The present findings uncover a new signalling element involved in the functionality of pilin-specific sortases controlling the pilus biogenesis of *Lb*. *rhamnosus* GG and related piliated Gram-positive species.

## Introduction

In Gram-positive bacteria, sortase enzymes play an essential role in displaying a large variety of effector proteins to the cell surface, allowing the bacterial cells to interact with their host and environment. Typically, sortases are either involved in anchoring proteins to the cell wall (the so-called housekeeping sortases) or in polymerizing pilin proteins (pilin-specific sortases) [1–3]. Due to their ability to display colonization or virulence factors, sortases have been widely investigated and constitute an alternative and promising target for antimicrobial treatment [4]. Sortases are not essential for bacterial cell survival but do significantly impact on the binding to host tissues, signaling to the host, or escaping the host immune response [5,6]. More recently, sortases have also been used to modify proteins *in vitro*, illustrating their use in synthetic biology [7].

Sortases are cysteine transpeptidases that attach proteins harboring a conserved pentapeptide motif (LP*X*TG) motif to another cell surface protein (polymerization reaction) or to peptidoglycan lipid II (anchoring reaction) [3]. Following the characterization of the first sortase enzyme Sa-SrtA and its gene in *Staphylococcus aureus* [8,9], genomic analysis revealed a remarkable diversity of sorting pathways, sequence motifs and sortase subfamilies [10]. In all studied systems, protein secretion and anchoring is a finely coordinated and regulated process that relies on multiple mechanisms and signaling pathways, the details of which are still emerging [11]. It has been recognized that the distribution of surface proteins on their cell walls differs between rod-shaped and coccoid cells. In *Streptococcus pyogenes*, proteins anchored to the cell wall are localized in separate foci in the vicinity of the septum [12], whereas in *Bacillus subtilis* proteins are secreted on the cell wall in a spiral shape along the cell axis [13]. In addition, the co-localization of the secretory machinery and sortases allow an efficient protein anchoring or assembly [14,15]. Sortase enzymes themselves harbor a number of conserved motifs essential for their specific localization. Thus, the lack of the positively charged C-terminal domain of *Enterococcus faecalis* sortases alter their distribution on the cell wall, resulting in the lack of pili [14]. Similarly, sortase-anchored proteins are retained in specific focal sites in the absence of sortases, suggesting that both sortases and their respective substrates are addressed to dedicated assembly sites, called ‘pilusosome’ [16]. It has been suggested that these foci are localized in the cell envelope regions abundant in lipid II, to which LP*X*TG proteins are ultimately cross-linked [17]. In *S*. *aureus*, the spatial distribution of the Sa-SrtA-anchored proteins is dependent on the presence of a conserved motif (YSIRK/GS) in their signal sequence and those with this motif are directed near the septum, whereas others lacking this motif are retained at peripheral foci [18]. Still, the complete secretory and anchoring machinery has been not fully comprehended and, more specifically, sortase trafficking and signaling systems have been poorly examined. The present study aims at identifying novel signal sequence elements within sortases involved in the translocation and secretion of the pilin-specific sortase enzymes in *Lactobacillus rhamnosus* GG. By comparing the pilus-specific sortases of well-characterized pilus systems in Gram-positive bacteria, we found a distinct N-terminal domain, termed here the GYPSY motif, located in the signal sequence. Hence, we identified the critical residues in the GYPSY motif in an experimental system based on pilus gene cassettes that were either expressed in *Lactococcus lactis* or a pilus-deficient *Lb*. *rhamnosus* derivative. The latter were examined by immunogold staining transmission electron microscopy, immunoblotting and mucus binding assays. The results show that the proline residue in the GYPSY motif is essential for the function of pilin-specific sortases and therefore pilus assembly in not only *L*. *rhamnosus* GG but potentially also in other piliated Gram-positive bacteria, which harbor this conserved GYPSY motif within the peptide signal of their pilin-specific sortases.

## Experimental Procedures

### Bioinformatics sequence analysis

The protein sequences of twenty-four pilin-specific sortases identified in 16 pilus gene clusters from 9 different Gram-positive bacterial species were obtained from public depository databases, including *i*.*e*. *Lactobacillus rhamnosus* GG [19], *Corynebacterium glutamicum* R [20], *Enterococcus faecium* TX0016 [21–23], *Bacillus cereus* ATCC 14579 [24,25], *Streptococcus agalactiae* 2603V/R [26–30], *Streptococcus pneumoniae* TIGR4 [31–33], *Enterococcus faecalis* V583 [34,35], *Corynebacterium diphtheriae* NCTC 13129 [36–38] and *Lactococcus lactis* IL1403 [39,40]. The sequence alignment of pilin-specific sortases was generated using MUSCLE [41]. The LOGO motif was generated using the MEME suite [42]. Further GYPSY motif search using KEGG database (http://www.genome.jp/tools/motif/) was performed. The input motif used for the motif search was GX_5_YPX_2_SX_2_Y, which corresponds to the motif initially identified in *Lb*. *rhamnosus* GG SrtC1. Search was restricted to prokaryotic KEGG database and proteins, where the motif start position was at most 30 amino acids from the start methionine. The secondary structure of GG SrtC1 was also analysed with PSIPRED [43,44] which showed the residues of the GYPSY motif to be part of a non-continuous helix. This motif was further modelled *ab initio* and the structural illustrations were generated using PyMOL software [45].

### Bacterial strains, plasmids, growth media and conditions

Bacterial strains and expression vectors used in the present study are shown in Table 1. *Lb*. *rhamnosus* strains GG and GG-PB12 (termed PB12 in the present work) were cultured in MRS broth in anaerobic conditions at 37°C. *Lb*. *rhamnosus* PB12 transformants were selected in MRS agar medium with 5 μg/ml chloramphenicol (Sigma Aldrich, MO, USA). *L*. *lactis* subsp. *cremoris* strain NZ9000 [46] and MG1363 derivatives were propagated anaerobically at 30°C in M17 broth (Oxoid, UK) with 0.5% (w/v) D-glucose (Sigma). *L*. *lactis* subsp. *cremoris* transformants were selected in M17 agar medium with 0.5% (w/v) D-glucose and 5 μg/ml chloramphenicol.

**Table 1:** Bacterial strains and plasmids used in the present study.

Strains or plasmids	Relevant properties and characteristics	References
Strains		
*E*. *coli* BL21-DE3	Expression host	NEB, MA, USA
*Lb*. *rhamnosus* GG	type strain (ATCC 53103)	Valio Ltd. culture collection
*Lb*. *rhamnosus* PB12	GG derivative strain, truncated *srtC1* gene	[47]
*L*. *lactis* NZ9000	MG1363 strain, *pep*N::*nis*R*nis*K genes	[46]
*L*. *lactis ppiA*	ppiA mutant of MG1363 by single crossing over, Em^R^	[48]
*L*. *lactis* Ctl (ppiA+)	control ppiA+ strain, Em^R^	[48]
Plasmids		
pET-SpaA	pET-52b(+) encoding a 798–bp *spa*A fragment, Amp^R^	This study
pNZ44	*L*. *lactis* expression vector, Cm^R^	[49]
pAC44	pNZ44 encoding *spa*A and *srt*C1	[11]
pGG44	pAC44 derivative, AA replacement SrtC1^Y29G, P30G^	This study
pYG44	pAC44 derivative, AA replacement SrtC1^P30G^	This study
pGP44	pAC44 derivative, AA replacement SrtC1^Y29G^	This study
pNZ44-SrtC1	pNZ44 encoding *srt*C1 gene	This study
pNZ44-SrtC1^Y29G, P30G^	pNZ44-SrtC1, AA replacement SrtC1^Y29G, P30G^	This study
pNZ44-SrtC1^P30G^	pNZ44-SrtC1, AA replacement SrtC1^P30G^	This study
pNZ44-SrtC1^Y29G^	pNZ44-SrtC1, AA replacement SrtC1^Y29G^	This study

Cm^R^, chloramphenicol resistance marker, Amp^R^, ampicillin resistance marker, Em^R^, erythromycin resistance

### Site-directed mutagenesis of the SpaA-SrtC1 pilus cassette

Oligonucleotide PCR primers were synthesized by Oligomer Oy (Helsinki, Finland). Expression vectors are shown in Table 1. To perform the site-directed mutagenesis, we amplified the plasmid pAC44 using back-to-back phosphorylated PCR oligonucleotides, as previously described [11]. The resulting PCR amplicon was then self-ligated using T4 DNA ligase (Promega). The ligation product was dialyzed and then electroporated into *L*. *lactis* electrocompetent cells [50]. Transformants were selected on M17 agar plates supplemented with 0.5% (w/v) D-Glucose and chloramphenicol (final concentration 5 μg/ml), screened and sequenced prior phenotypic analysis. Similar procedure was performed for pGG44, pYG44 and pGP44.

### Complementation of pilus-less *Lb*. *rhamnosus* PB12

The full-length *srtC*1 gene was cloned into pNZ44 [49] resulting in the construct pNZ44-SrtC1. Briefly, the *srtC1* gene was amplified by PCR using Phusion Hot Start II High-Fidelity DNA Polymerase (Thermo Fischer Scientific, PA, USA) and flanked with NcoI and SpeI restriction sites and a ribosome binding site (RBS). Both insert (*srtC*1 gene amplicon) and pNZ44 plasmid were digested by NcoI and SpeI (FastDigest restriction enzymes, Thermo Fischer Scientific) for 30 min at 37°C and then ligated overnight at 4°C with T4 DNA ligase (Promega). The ligation product was then cloned into *L*. *lactis* NZ900 as detailed above. Three additional derivatives pNZ44-SrtC1^Y29G,P30G^, pNZ44-SrtC1^P30G^ and pNZ44-SrtC1^Y29G^ were generated using the same mutagenesis approach as described above. The correct construction of all four vectors was verified by DNA sequencing analysis. Next, electrocompetent *Lb*. *rhamnosus* PB12 were prepared and transformed with pNZ44-SrtC1, pNZ44-SrtC1^Y29G,P30G^, pNZ44-SrtC1^P30G^ and pNZ44-SrtC1^Y29G^ following the same protocol as in a previous study [51].

### Production of polyclonal antibodies directed against SpaA pilin protein

A 798 bp-long fragment of the *spaA* gene was codon-optimized, *in vitro* synthesized and cloned into pET-52b(+) (Merck Millipore, Germany) by GenScript USA Inc. (NJ, USA). The plasmid was then introduced into *E*. *coli* BL21-DE3 (NEB, MA, USA) and the SpaA fragment was produced as described in the QIAexpressionist manual (Qiagen, Germany). SpaA protein was purified using PrepEase Histidine-tagged Protein Purification Midi Kit (Affimetrix, CA, USA) and desalted using Econo-Pac 10DG Desalting Columns (Biorad, CA, USA). Following concentration using Amicon Ultra-4 Centrifugal Filter Unit with Ultracel-10 membrane (Merck Millipore, Germany), the SpaA protein was analysed by protein gel electrophoresis and quantified by NanoDrop (Thermo Fischer Scientific). Rabbit immunization procedures were performed at the Laboratory Animal Centre (Large Animal Unit) of the University of Helsinki (Finland) [52]. Terminal bleeds were collected and the SpaA antiserum was aliquoted and stored at -80°C for further use.

### Cell-wall extraction and Western blotting analysis

*Lb*. *rhamnosus* cell wall extracts were prepared as previously described [11,53] and then examined by Western-blotting analysis [54] using polyclonal anti-SpaA rabbit antibodies (dilution 1/ 25,000) and goat anti-rabbit IgG (H + L)-horseradish peroxidase conjugate (dilution 1/100,000, Biorad) as primary and secondary antibodies, respectively.

### Immunogold staining transmission electron microscopy

Bacterial cells harvested from overnight cultures were immunogold-stained using rabbit polyclonal anti-SpaA antibodies (dilution 1/100) and gold particule (10 nm) conjugated protein A (dilution 1/55) and then immobilized on copper grids as described before [55]. Sample grids were observed using JEM-1400 transmission electron microscope (JEOL Ltd., Japan).

### Mucus binding assays

Mucus adhesion assays of the different *Lb*. *rhamnosus* strains were performed, as described previously [47,56]. The ratios between the bound bacterial cells and the total bacterial cells resulted in percentages of binding to pig stomach type II mucus (Sigma Aldrich, MO, USA) and were based on twelve technical replicates of three biological repeats.

## Results

### *In silico* analysis of the N-terminal domain of pilin-specific sortases from different piliated Gram-positive bacteria

**Fig 1:**
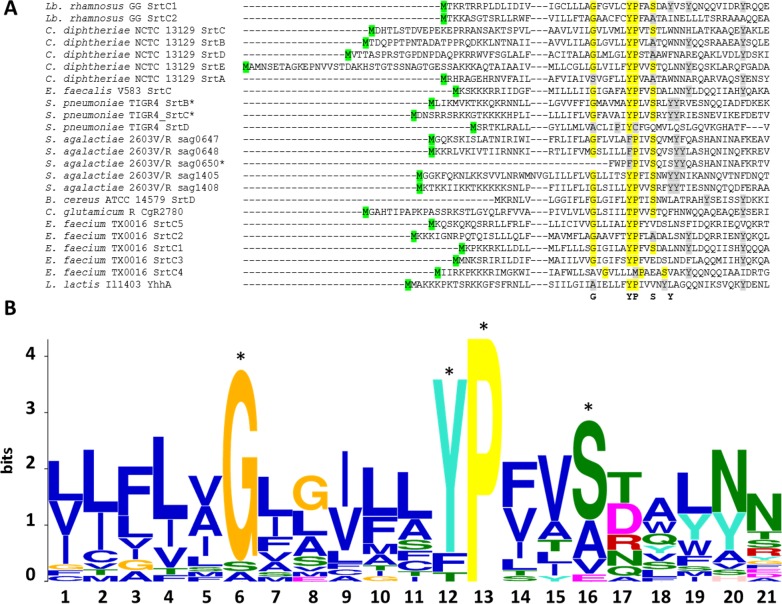
Sequence alignment of the N-terminal domains of pilin-specific sortases from various piliated Gram-positive species and LOGO motif analysis. (A) The alignment was generated using MUSCLE [41]. The so-called GYPSY motif is highlighted in yellow, with the tyrosine residue and the proline residue constituting the functional core of the motif. Residues with a partial degree of conservation within the GYPSY motif are shaded in light grey. The start methionine is highlighted in green. Protein sequences were obtained from public databases, as indicated in the text. In the table, the start of the three protein sequences marked with an asterisk (*) were re-analyzed in the present study. In addition, all sortase sequences excluding *E*. *faecium* SrtC4, *S*. *pneumoniae* TIGR4 SrtD and *S*. *agalactiae* 2603V/R sag0650 were analyzed using the MEME suite [42], resulting in the LOGO motif depicted in (B). Legend: blue, hydrophobic residue; green, polar, non-charged residue; magenta, acidic residue and red, positively charged residue.

**Fig 2:**
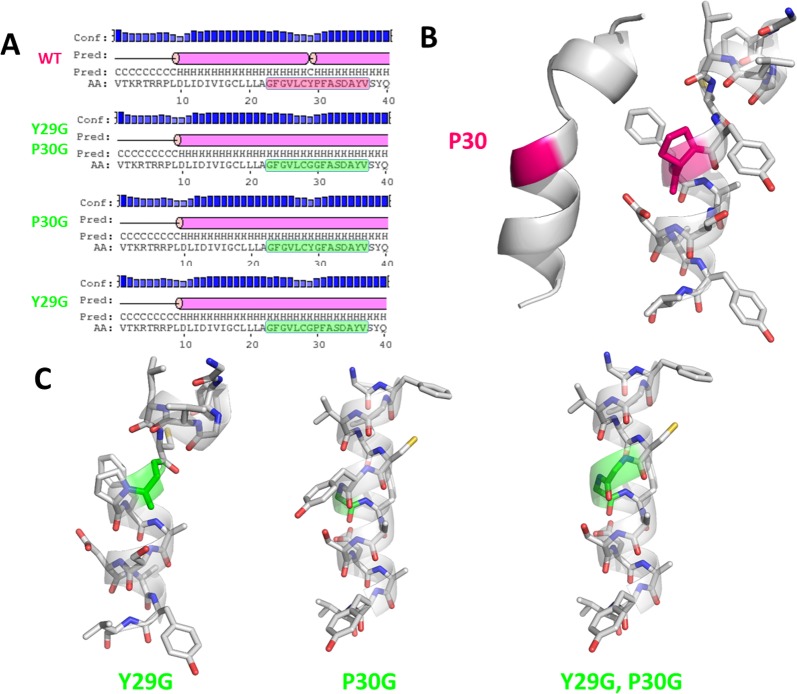
Modelling of the region containing the GYPSY motif of the *Lb*. *rhamnosus* GG SrtC1 with kinked proline residue. (A) Output prediction results using PSIPRED server [43,44] indicated that the GYPSY motif introduces a breakage within an α-helix as opposed to some mutant variants; (B) and (C), *ab initio* model of the GYPSY motif. The kinked proline residue is shaded in fuchsia. The relevant AA modification(s) introduced in the present study are shown in vivid green.

The sequences of twenty-four pilin-specific sortases from 16 different pilus gene clusters were analysed and revealed the conservation of residues located within the peptide signal sequence, termed the GYPSY motif (Fig 1). It generally consists of the following consensus sequence GX_5_YPX_2_SX_n_Y, n⊰[2;6]. More specifically, the juxtaposition of the tyrosine (Y29) residue and the proline (P30) residue was found in all analysed sequences, except in *S*. *pneumoniae* TIGR4 SrtD and *E*. *faecium* TX0016 SrtC4. In the latter, a methionine (M) residue was present instead of the first tyrosine (Y) residue. TX0016 SrtC4 has been reported to belong to a pilus gene cluster located on a megaplasmid [57], possibly resulting in a high degree of sequence polymorphism due to the mobility potential of such DNA element. In *S*. *agalactiae* strain 2603V/R, one of its two pilus gene clusters also harboured a set of three pilin-specific sortases all having a phenylalanine (F) residue replacing the first tyrosine (Y) residue. In all 16 pilus gene clusters, there is a high diversity in terms of gene order. However, the GYPSY motif was widely conserved, indicating that it may play a similar role in these species, possibly in the translocation and secretion of pilin-specific sortases to the cell wall. Additional sequence searches further identified the GYPSY motif in a larger number of pilin-specific sortase from strains belonging to *Enterococcus*, *Lactobacillus* and *Streptococcus* spp. Remarkably, the analyzed housekeeping sortases did not have such a GYPSY motif within their N-terminal domains, as observed for example in *Lb*. *rhamnosus* GG, *L*. *lactis* MG1363, *E*. *faecalis* V583 or *S*. *agalactiae* 2603V/R genomes (data not shown). Additional bioinformatics analysis using Motif Search for the GX_5_YPX_2_SX_2_Y query sequence revealed that proteins harboring the latter motif within the first 30 amino acid of the N-terminal domain were prevalently annotated as sortases (S1 Fig), further illustrating that such motif within the signal peptide is somehow characteristic to sortases. Secondary structure prediction of the *Lb*. *rhamnosus* SrtC1 GYPSY motif and *ab initio* modelling revealed that the GYPSY motif forms a broken α-helix (Fig 2). Proline has the particularity to introduce kink [58], impacting on the geometry and therefore biological functions [59–61]. Substitution of the proline (P30) residue by a glycine (G) residue is predicted to remove the kink induced by the presence of the proline residue thus forming an uninterrupted helix, as illustrated in SrtC1^Y29G,P30G^ and also in SrtC1^P30G^ (Fig 2). Although the secondary structure of SrtC1^Y29G^ was predicted to be an uninterrupted helix by PSIPRED (Fig 2), we concluded that the helix in the GYPSY motif may be also broken owing to the presence of the proline (P30) as in the wild-type SrtC1 sequence and as suggested by the mutagenesis experiments detailed below. We hypothesized that mutant variants SrtC1^Y29G,P30G^ and SrtC1^P30G^ may have an impact on pilus formation. Experimental models used in the present study aimed at determining the functional roles of each these residues in regard with our hypothesis and predicted models.

With regard to the distinct functions of pilin-specific sortases (polymerization) and housekeeping sortases (anchoring), we hypothesized that the GYPSY motif, and intrinsically its conformation, may be functionally important for the stability, translocation and/or secretion of the pilin-specific sortases. This would then constitute an additional regulatory pathway, controlling the pilus assembly in Gram-positive species. We used site-directed mutagenesis together with biochemical, microscopic and complementary microbiological analysis methods to test this hypothesis using the rod-shaped and coccoid model systems derived from *Lb*. *rhamnosus* GG and *Lactococcus lactis* subsp. *cremoris* strains MG1363 and NZ9000.

### Alteration of the peptide signal impacts on piliation in a lactococcal model

**Fig 3:**
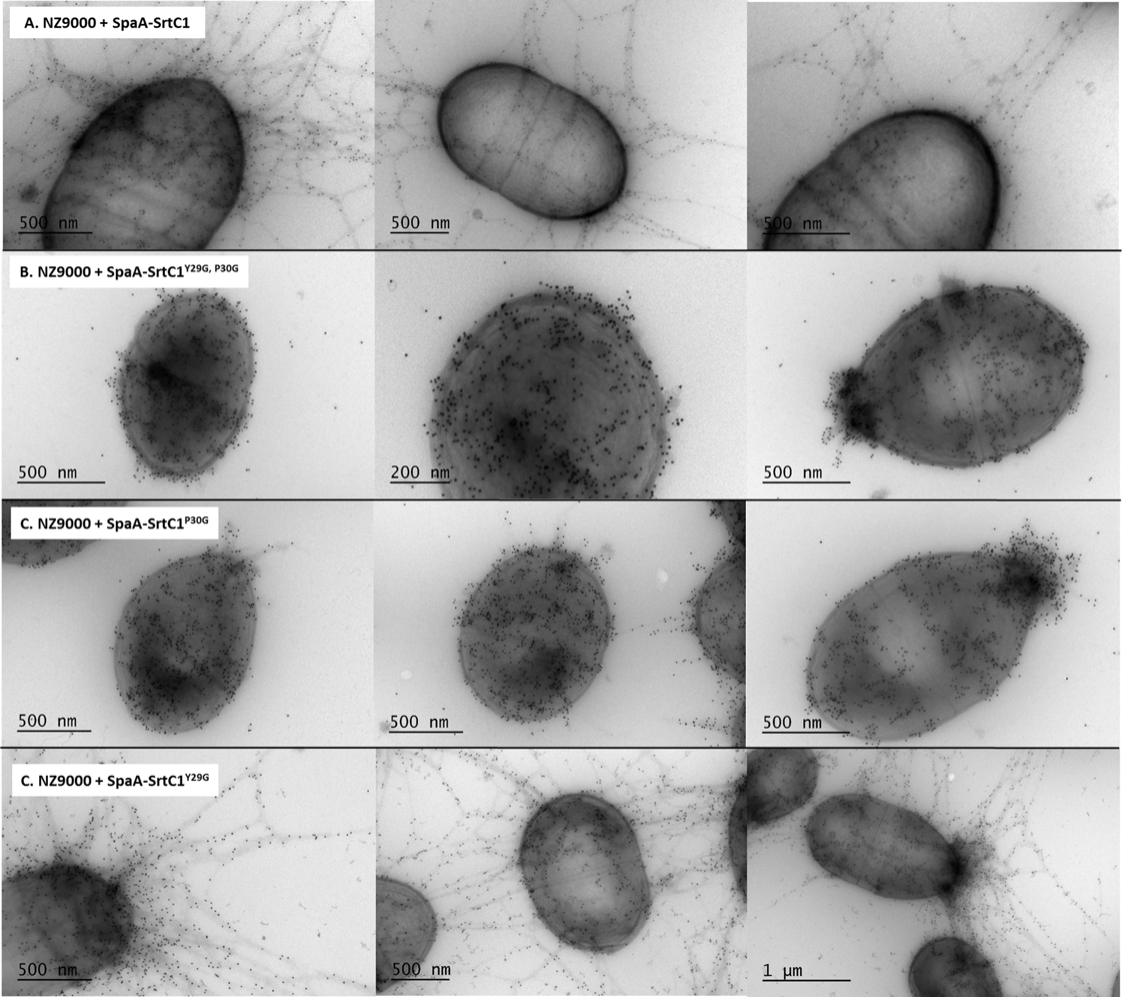
Electron microscopy observations of *L*. *lactis* NZ9000 producing various SpaA pilus structures. Bacterial cells were immunogold-labeled with anti-SpaA serum and gold particles (10 nm). Legend: A, NZ9000 + SpaA-SrtC1; B, NZ9000 + SpaA-SrtC1^Y29G, P30G^; C, NZ9000 + SpaA-SrtC1^P30G^; D, NZ9000 + SpaA-SrtC1^Y29G^.

**Fig 4:**
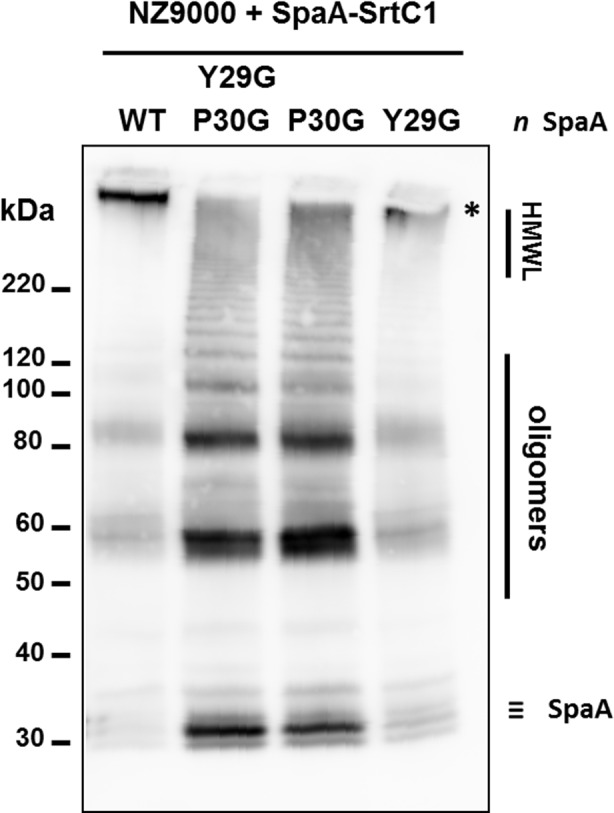
Western blotting analysis of *L*. *lactis* NZ9000 expressing different *spaA-srtC*1 gene variants, where the GYPSY motif has been altered by AA substitution. **SpaA pilin proteins were detected using anti-SpaA polyclonal antibodies.** Legend: #, protein weight marker; HMWL, high molecular weight ladder; *, band corresponding to elongated pilus structures.

In previous work, we expressed the *Lb*. *rhamnosus* GG *spaA*-*srtC*1 in *L*. *lactis* NZ9000 to investigate the signalling pathway involved in sortase specificity during pilus biogenesis [11]. Using the same lactococcal model system complemented with a plasmid encoding the SpaA major pilin subunit and the pilin-specific sortase SrtC1, we specifically mutated the tyrosine (Y29) and proline (P30) residues of the GYPSY motif of SrtC1. The GYPSY motif is highly conserved in pilin-specific sortases, including the ones found in *L*. *lactis* (Fig 1) and absent in its housekeeping sortases analyzed, possibly indicating that it may constitute an original signalling in pilin-specific sortase translocation. In contrast with NZ9000 producing SpaA and SrtC1 (positive control), the replacement of both tyrosine (Y29) and proline (P30) residues by glycine (G) residues within the GYPSY motif of SrtC1 reduced the degree of SpaA piliation in *Lactococcus lactis* cells. Detailed analysis by immune-electron microscopy showed SpaA pilin subunits to appear predominantly on the cell wall in a monomeric or oligomeric form (Figs 3 and 4). Interestingly, in a small fraction of the *L*. *lactis* NZ9000 population producing SpaA-SrtC1^Y29G,P30G^, some SpaA aggregates also accumulated at the poles of the cells (Fig 3C). This peculiar phenotype was, however, observed in only few cells. The SrtC1 Y29G substitution did not significantly impact on pilus assembly in *L*. *lactis* NZ9000, whereas the SrtC1 P30G substitution impaired pilus assembly. Few cells displaying short pili and SpaA aggregates could also be observed at the cell poles. Immuno-electron microscopy observations in *L*. *lactis* mutant derivatives indicated that the proline (P30) residue had a significant impact on the functionality of SrtC1 and therefore negatively affects the assembly of pili. We did not address whether the alteration of the peptide signal (especially the GYPSY motif) has a deleterious effect on the stability of SrtC1 and/or the translocation/secretion of SrtC1 to the cell wall of *L*. *lactis* NZ9000. Nevertheless, it seems reasonable to believe that the substitution of 1 or 2 amino-acid residues is not likely to impact on the overall stability of the whole protein SrtC1 but rather on some associated mechanisms that required specific and well-conserved motifs.

### Complementation of pilus-less *Lb*. *rhamnosus* PB12 and restoration piliation

**Fig 5:**
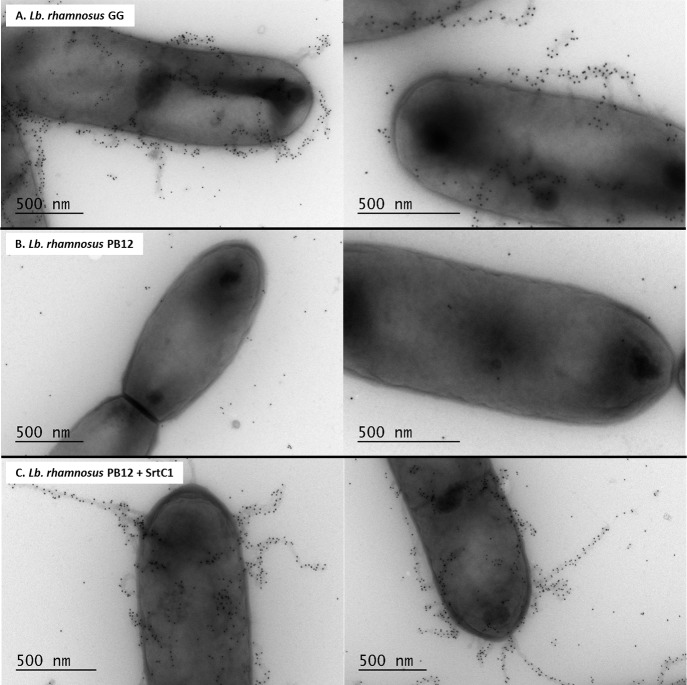
Electron microscopy observations of *Lb*. *rhamnosus* PB12 and its SrtC1-complemented derivatives. Bacterial cells were immunogold-labeled with anti-SpaA serum and gold particles (10 nm). Legend: A, *Lb*. *rhamnosus* GG; B, *Lb*. *rhamnosus* PB12; C, *Lb*. *rhamnosus* PB12 + SrtC1.

**Fig 6:**
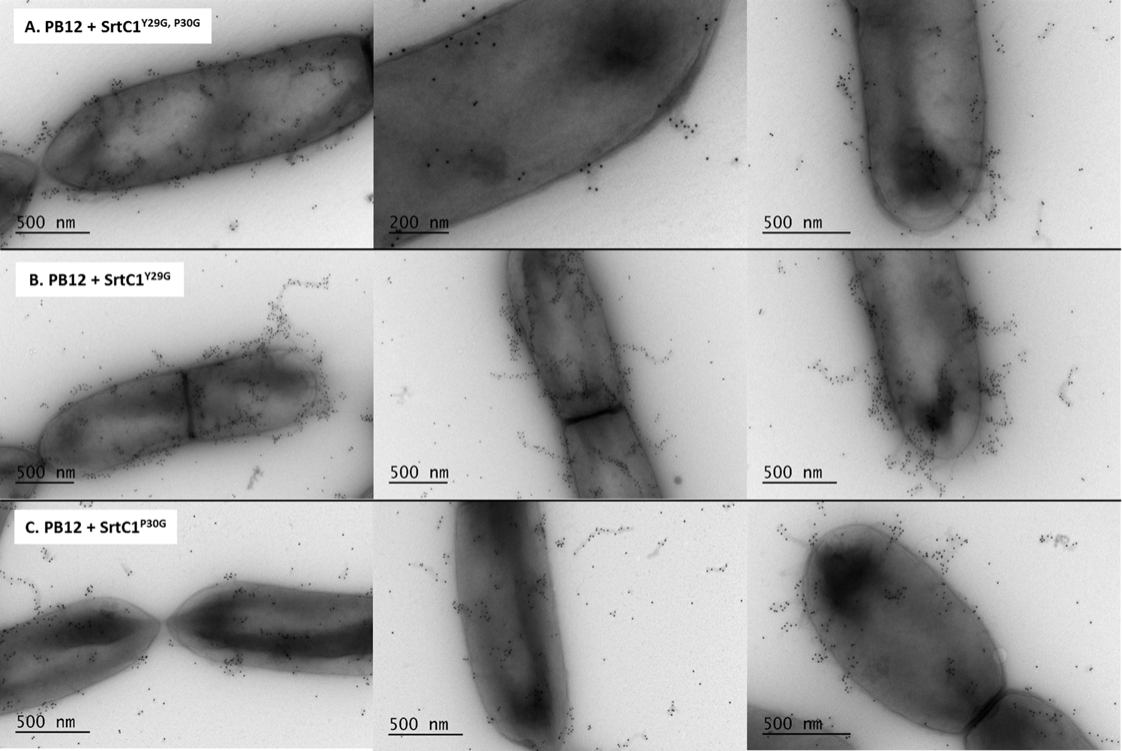
Electron microscopy observations of *Lb*. *rhamnosus* PB12 complemented with SrtC1 variants. Bacterial cells were immunogold-labeled with anti-SpaA serum and gold particles (10 nm). Legend: A, *Lb*. *rhamnosus* PB12 complemented with SrtC1 ^Y29G, P30G^; B, *Lb*. *rhamnosus* PB12 complemented SrtC1 ^Y29G^; C, *L*. *rhamnosus* PB12 complemented SrtC1 ^P30G^.

**Fig 7:**
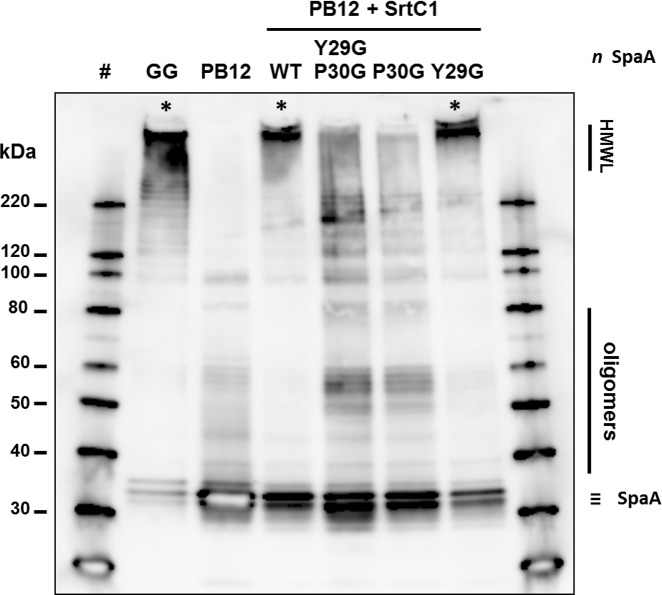
Western blotting analysis of *Lb*. *rhamnosus* GG and PB12 expressing different *srtC*1 gene variants, where the GYPSY motif has been altered by AA substitution. SpaA pilin proteins were detected using anti-SpaA polyclonal antibodies. Legend: #, protein weight marker; HMWL, high molecular weight ladder; *, band corresponding to elongated pilus structures.

**Fig 8:**
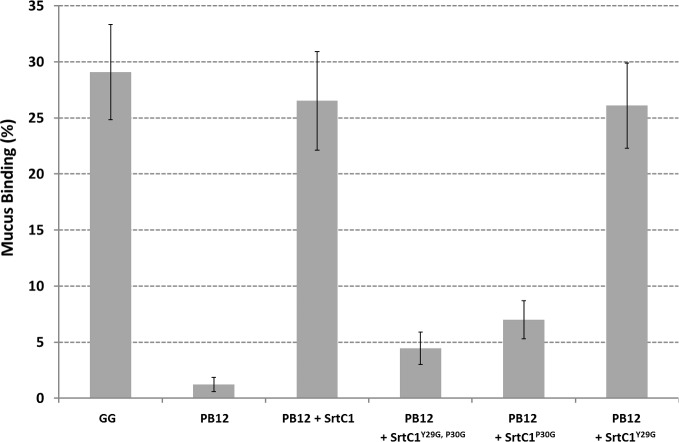
Mucus binding assays of *Lb*. *rhamnosus* GG and PB12 expressing different *srtC*1 gene variants, where the GYPSY motif has been altered by AA substitution. The averages and standard deviations were obtained from a total of twelve technical replicates from three biological replicates.

The pilus-less *Lb*. *rhamnosus* PB12 is a well-characterized, non-GMO derivative of strain GG that harbors a truncated *srtC*1 gene due to the presence a stop codon within the *srtC*1 open reading frame [47]. We used this strain to further investigate the GYPSY motif by complementing it with different SrtC1 variants. The introduction of the full-length *srtC*1 gene into PB12 restored the piliation, as observed by electron microscopy (Fig 5). This confirmed that the pilus-less phenotype observed in PB12 was only due to the truncation of SrtC1 and not any of the other collateral chromosomal mutations reported [47]. Numerous and elongated pilus structures could be observed in the complemented PB12 in contrast to PB12, which was devoid of any pili. The complementation of PB12 with SrtC1 mutant variants further supported the initial observations done in the lactococcal model. The introduction of either SrtC1^Y29G,P30G^ or SrtC1^P30G^ into *Lb*. *rhamnosus* PB12 did not result in wild-type piliation but rather in SpaA oligomers (Fig 6). The SrtC1 Y29G substitution, however, did not prevent the pilus assembly and resulted in a phenotype similar to *Lb*. *rhamnosus* GG, as observed by EM. Immunoblotting analysis using polyclonal SpaA antibodies also showed that PB12 complemented with SrtC1 or SrtC1^Y29G^ harboured long pilus structures (high molecular weight ladder) similarly to the piliated type strain *Lb*. *rhamnosus* GG (Fig 7). In complemented strains PB12 with SrtC1^Y29G,P30G^ or SrtC1^P30G^, SpaA oligomers could be observed and the degree of high molecular weight SpaA multimers was lower. In contrast, in GG and also PB12 with SrtC1 and PB12 with SrtC1^Y29G^, we were able to observe a higher degree of piliation, as indicated with an asterisk (*). It is noteworthy that the different SrtC1 protein variants are clearly expressed, since immunoblotting data (Fig 7) do show pilin polymerization or at least some degree of pilin oligomerization in the different mutants, as opposed to the negative control PB12, which is devoid of functional sortase SrtC1. In the latter (PB12), only SpaA monomers are produced. To further support our data and provide more quantitative measures of the degree of piliation, we measured the mucus binding ability of the different strains using an *in vitro* binding assay, where we measured the fraction of mucus-bound radiolabelled cells from the total amount of cells applied to the mucus-coated wells (Fig 8). *Lb*. *rhamnosus* PB12 complemented with either SrtC1 or SrtC1^Y29G^ displayed a high mucus binding capacity, which was almost at comparable levels with that of the wild-type *Lb*. *rhamnosus* GG. In contrast, the introduction of SrtC1^Y29G,P30G^ and SrtC1^P30G^ into strain PB12 did not significantly increase the mucus binding ability, indicating that SrtC^Y29G,P30G^ and SrtC1^P30G^ are not fully functional. Immuno-electron microscopy data, immunoblotting analysis and mucus binding assays were all concordant and showed that the substitution of the proline (P30) residue within the GYPSY motif impaired directly or indirectly the pilus assembly. As also observed in the results obtained in our lactococcal model, the reduction of SrtC1 activity remains to be identified, but we hypothesized that the substitution of 1 or 2 amino acid residues did not impact on the stability and expression of the different variants SrtC1, but rather on some specific mechanisms relating to the translocation of SrtC1 to the cell membrane.

### Expression of SpaA-SrtC1 in *L*. *lactis* MG1363 ppiA mutant

**Fig 9:**
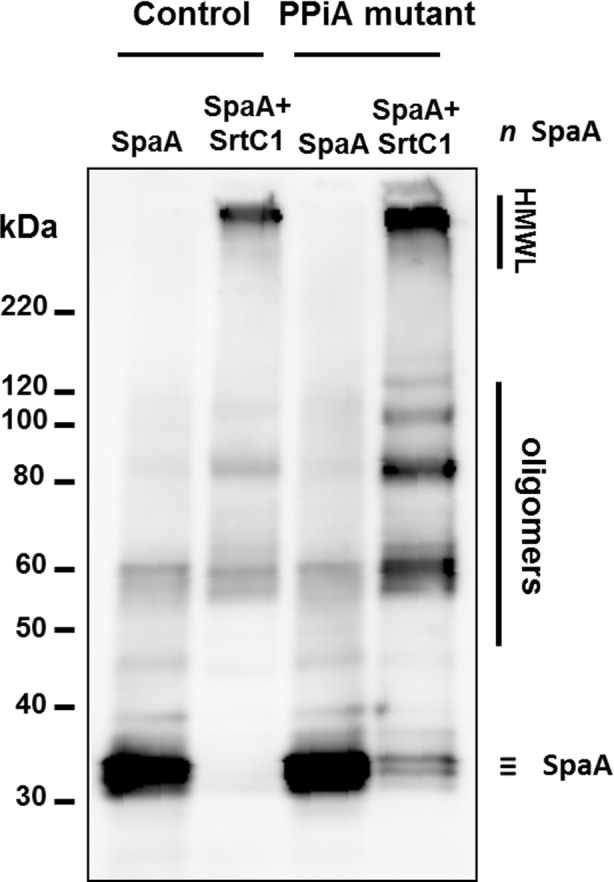
Western blotting analysis of *L*. *lactis* MG1363 PPiA KO mutant and its complemented mutant, where the SpaA-SrtC1 cassette was introduced. SpaA pilin proteins were detected using anti-SpaA polyclonal antibodies. Legend: #, protein weight marker; HMWL, high molecular weight ladder; *, band corresponding to elongated pilus structures.

The identification of the GYPSY motif in the SrtC1 peptide signal and its effect on pilus biogenesis highlighted an important role of the proline residue (P30) in the functionality of pilin-specific sortase SrtC1. In Fig 2, we showed that the proline (P) residue introduce a breakage of the left-hand α-helix and hypothesized that the proline kink is conformationally required to ensure stability and/or translocation of SrtC1 to the cell wall. It is known that *cis-trans* isomerization of the proline (P) by prolyl peptidyl isomerase (PPI) may also play a role in protein folding kinetics, geometry and biological functions. To further verify this, the pilus cassette encoding SpaA-SrtC1 was then electroporated into *L*. *lactis* MG1363 *ppiA* mutant and its control derivative [48] (Table 1). The *L*. *lactis* MG1363 *ppiA* mutant has no functional prolyl peptidyl isomerase, preventing any *cis-trans* isomerization transition. Pilus phenotype was analyzed by immunoblotting blotting (Fig 9) and revealed that the *L*. *lactis ppiA* mutant did produce SpaA pili, as also observed in the control, suggesting that the prolyl peptidyl isomerase ppiA is not involved in the stability and/or translocation of SrtC1 and therefore not required for pilus biogenesis as tested in a lactococcal model.

## Discussion

In contrast with other piliated Gram-positive species studied so far, *Lb*. *rhamnosus* GG has been shown to confer health promoting properties for the host and therefore has been widely used in food industry [62–65]. The identification of mucus-binding pili in GG [19] has opened the way to address their role in the probiotic and host signalling response [45]. Much of the research that has been done on the *Lb*. *rhamnosus* pilus biogenesis [11,54,55], showed some similarities in terms of function, assembly or gene order with other pilus clusters from Gram-positive piliated species and more specifically lactobacilli [66]. The present study expanded on this and provided new insight into the signalling pathways involved in the pilus assembly in *Lb*. *rhamnosus* GG. We showed that the GYPSY motif is highly conserved in pilin-specific sortases in Gram-positive piliated species.

We, therefore, proposed that the presence of the GYPSY motif in these bacterial species is likely to play a role in regard with the functionality of pilin-specific sortases that control the biogenesis of pili. Specifically, the proline residue is found in most sortase protein sequences analysed, possibly indicating its role in the conformation of the peptide signal of pilin-specific sortases in *Lb*. *rhamnosus* but also *C*. *diphtheriae*, *E*. *faecium* or *S*. *pneumoniae*. Other residues of the GYPSY motif did show a higher degree of diversity, such the tyrosine (Y) residue, which was replaced by a phenylalanine (F) residue in all sortases of one pilus gene cluster from *S*. *agalactiae* 2603V/R or by a methionine (M) residue in the SrtC4 from *E*. *faecium* TX0016. The tyrosine (Y) residue juxtaposing the proline (P) residue may be therefore not functionally important. Using mutagenesis, this was confirmed in both *L*. *lactis* and *Lb*. *rhamnosus*, where the P30G substitution had the highest impact on the degree of piliation, whereas the Y29G substitution did not. Other small residues constituting the GYPSY motif (G23 and S33) were not investigated but may have a limited role since they seem to be less conserved.

Phenotype analysis indicated that the loss of the proline (P30G) residue reduced the pilus assembly and only SpaA monomers and aggregates could be visualized by EM and immunoblotting in *L*. *lactis* (Table 2). Remarkably, a subpopulation of *L*. *lactis* NZ9000 cells (expressing SpaA-SrtC1^Y29G,P30G^ or SpaA-SrtC1^P30G^) display an accumulation of SpaA-mers and few short pili at the poles of the cells. We hypothesized that such phenotype may be possibly attributed to the poor stability/translocation of SrtC1, resulting in the assembly of few short and aborted pili, which are then moved away from the septum upon growth and division of the bacterial cells. In *Lb*. *rhamnosus*, we also demonstrated that the GYPSY motif and more specifically the proline (P30) residue was important for SrtC1 functionality and indirectly for pilus assembly (Table 2). Its location within the peptide signal further suggests that the proline may determine a specific conformation of the peptide signal, directly relating to the secretion-competent state or stability of the pilin-specific sortase SrtC1. The close vicinity of the GYPSY motif with the predicted peptide signal cleavage (S33) suggests a possible link between peptide signal, stability and translocation of SrtC1. Proline residue confers unique properties to protein structure and we proposed that the presence of a proline kink is functionally important, although we do not currently know if it has an effect on SrtC1 stability or translocation. We also ruled out the possibility of a prolyl peptidyl isomerase-mediated regulatory pathway (*cis-trans* isomerization), since we showed that prolyl peptidyl isomerase was not required for the production of pili. It, therefore, remains to be determined what are the regulatory controls and mechanisms associated with the GYPSY motif, *i*.*e*. protein translocation, stability or chaperone activity. Peptide signals are expected to be functionally important for the specific and coordinated addressing and translocation of pilin subunits and sortases to the pilusosomes (assembly sites). The recent description of a signal peptidase involved in processing secreted proteins suggests that a number of players remains to be identified with regard to their respective role in pilin and sortase targeting [67]. The present study is the first report that described the presence of proline within the peptide signal, as a critical signal element for the functionality of pilin-specific sortases.

**Table 2:** Summary of pilus phenotypes observed in derivatives of *Lactococcus lactis* NZ9000, *Lactobacillus rhamnosus* GG and PB12. The AA replacement is shaded in light grey within the GYPSY motif (bold AA residues).

Bacterial Derivatives	GYPSY Motif	Pili Assembly	Description of Pilus Phenotype
*Lactobacillus rhamnosus* GG	**G**FGVLC**YP**FA**S**DAY	+	High molecular weight pili
*Lactococcus lactis* NZ9000+pAC44			
- wild-type SrtC1	**G**FGVLC**YP**FA**S**DAY	+	High molecular weight pili
- SrtC1^Y29G, P30G^	**G**FGVLC**GG**FA**S**DAY	+/-	SpaA multimers
- SrtC1^Y29G^	**G**FGVLC**GP**FA**S**DAY	+	High molecular weight pili
- SrtC1^P30G^	**G**FGVLC**YG**FA**S**DAY	+/-	SpaA multimers
*Lactobacillus rhamnosus* PB12			
- negative control	n/a	-	No pili, SpaA monomers
- complemented with wild-type SrtC1	**G**FGVLC**YP**FA**S**DAY	+	High molecular weight pili
- complemented with SrtC1^Y29G, P30G^	**G**FGVLC**GG**FA**S**DAY	+/-	SpaA multimers
- complemented with SrtC1^Y29G^	**G**FGVLC**GP**FA**S**DAY	+	High molecular weight pili
- complemented with SrtC1^P30G^	**G**FGVLC**YG**FA**S**DAY	+/-	SpaA multimers

Legend: +, presence of pili; -, absence of pili; +/-, presence of short and/or rare pili; n/a, not applicable.

The structures of housekeeping and pilin-specific sortases have been solved in a number of species [68]. Unfortunately, very little is known about the detailed conformation of peptide signals of these as these have not been included in the solved crystal structures. Their hydrophobic propensity prevents their structural studies, leaving us with *in silico* prediction to understand their structure and function. Due to the pivotal role of sortases in displaying interaction factors in pathogenic and non-pathogenic bacteria, a further understanding of the mechanisms governing the translocation and maturation of sortases would offer new approaches in the development of anti-microbial inhibitors blocking the interaction capabilities of bacteria towards the host.

## Supporting Information

S1 Fig GYPSY motif search in KEGG database using MOTIF Search (http://www.genome.jp/tools/motif/). The input motif used for the search was the following one: G-x-x-x-x-x-Y-P-x-x-S-x-x-Y, which corresponds to the motif identified in *L*. *rhamnosus* GG SrtC1. We restricted our search to prokaryotic KEGG gene database and only considered proteins, where the motif start position was at most 30 amino acids from the start methionine. Out of 685 hits found in the prokaryotic KEGG gene database, 87 of them did have the GX_5_YPX_2_SX_2_Y motif located at the N-terminal domain. Interestingly, 69 out of 87 were proteins annotated as sortase enzymes. (A) The list below shows the complete list of proteins found through this search. (B) Using these 69 sequences, we also generated the LOGO motif using the MEME Suite.
(PDF)
