# Supplementary material for: The N-Terminal GYPSY Motif Is Required for Pilin-Specific Sortase SrtC1 Functionality in Lactobacillus rhamnosus Strain GG
Source: PLoS One. 2016 Apr 12;11(4):e0153373. doi: 10.1371/journal.pone.0153373 (PMC4829219; doi:10.1371/journal.pone.0153373)

Panel A

Species	Strain	Gene Name	Sequence
Paenibacillus borealis	DSM 13188	PBOR_03375	MRKLSYLIILAGVLIMLYPKGSEWYNDRQQQKLLEEAEQAYSEIVPSASAPAGADLSKPY
Paenibacillus sp.	FSL P4-0081	P40081_03675	MRKLSYLIILAGVLIMLYPKGSEWYNDRQQQKLLEEAEQAYSEIVPSASAPASADLSKPY
Paenibacillus sp.	FSL R5-0912	R50912_03350	MRKLSYLIILAGVLIMLYPKGSEWYNDRQQQKLLEEAEQAYSEIVPSASAPASADLSKPY
Paenibacillus sp.	FSL R7-0273	R70723_02905	MRKLSYLIILAGVLIMLYPKASEWYNDRQQAKLLEEAEQAYSELAPAPGPDLQKPYAEVT
Paenibacillus sp.	FSL H7-0357	H70357_03230	MRKFSYLIIIAGVLVMLYPKASEWYNDRQQEKLLKEAEQAYSELAPSSEPDLKKQYAEVT
Streptococcus pyogenes	MGAS10750 (serotype M4)	MGAS10750_Spy0118	MKKQSKRHRLNQNIALIVVFLIGLTILLYPQISRIYYTIESNYQSKQFDREKSTLHQEDI
Streptococcus pyogenes	MGAS10750 (serotype M4)	MGAS10750_Spy0119	MLKNKQSLSLRERLIQVMFPLLFLMGCLIVLYPLMSNYYYRVKQNQAVTSFESAKVIVNK
Streptococcus pneumoniae	Hungary19A 6	SPH_0576	MVKTKKQKRNNLLLGVVFFIGMAVMAYPLVSRLYYRVESNQQIADFDKEKATLDEADIDE
Streptococcus pneumoniae	Hungary19A 6	SPH_0577	MDNSRRSRKKGTKKKKHPLILLLIFLVGFAVAIYPLVSRYYYRIESNEVIKEFDETVSQM
Streptococcus pneumoniae	Taiwan19F-14	SPT_0505	MAKTKKQKRNNLLLGVVFFIGMAVMAYPLVSRLYYRVESNQQIADFDKEKATLDEADIDE
Streptococcus pneumoniae	Taiwan19F-14	SPT_0506	MVGLAVAMYPLVSRYYYRIESNEVIKEFDETVSQMDKAELEERWRLAQAFNATLKPSEIL
Streptococcus pneumoniae	TCH8431/19A	HMPREF0837_10762	MAKTKKQKRNNLLLGVVFFIGMAVMAYPLVSRLYYRVESNQQIADFDKEKATLDEADIDE
Streptococcus pneumoniae	TCH8431/19A	HMPREF0837_10763	MSKSRYSRKKSVKKKKNPFILLLIFLVGLAVAMYPLVSRYYYRIESNEVIKEFDETVSQM
Streptococcus pneumoniae	670-6B	SP670_0544	MVKTKKQKRNNLLLGVVFFIGMAVMAYPLVSRLYYRVESNQQIADFDKEKATLDEADIDE
Streptococcus pneumoniae	ST556	MYY_0537	MKKKKNPFILLLIFLVGLAVAMYPLVSRYYYRIESNEVIKEFDETVSQMDKAELEERWRL
Streptococcus pneumoniae	A026	T308_02250	MAKTKKQKRNNLLLGVVFFIGMAVMAYPLVSRLYYRVESNQQIADFDKEKATLDEADIDE
Streptococcus pneumoniae	A026	T308_02255	MSKSRYSRKKSVKKKKNPFILLLIFLVGLAVAMYPLVSRYYYRIESNEVIKEFDETVSQM
Streptococcus agalactiae	2603 (serotype V)	SAG1405	MGGKFQKNLKKSVVLNRWMNVGLILLFLVGLLITSYPFISNWYYNIKANNQVTNFDNQTQ
Streptococcus agalactiae	2603 (serotype V)	SAG1406	MKTKKIIKKTKKKKKSNLPFIILFLIGLSILLYPVVSRFYYTIESNNQTQDFERAAKKLS
Streptococcus agalactiae	09mas018883	BSA_14860	MGGKFQKNLKKSVVLNRWMNVGLILLFLVGLLITSYPFISNWYYNIKANNQVTNFDNQTQ
Streptococcus agalactiae	09mas018883	BSA_14870	MKTKNINKKTKKKKSNLPFIILFLIGLSILLYPVVSRFYYTIESNNQTQDFERAAKKLSQ
Streptococcus agalactiae	NGBS061	EN72_07795	MGGRFQKNLKKSVVLNRWMNVGLILLFLVGLLITSYPFISNWYYNIKANNQVTNFDNQTQ
Streptococcus agalactiae	NGBS061	EN72_07800	MKTKNIIKKTKKKKSNLPFIILFLIGLSILLYPVVSRFYYTIESNNQTQDFERAAKKLSQ
Streptococcus agalactiae	CNCTC 10/84	W903_1421	MGGRFQKNLKKSVVLNRWMNVGLILLFLVGLLITSYPFISNWYYNIKANNQVTNFDNQTQ
Streptococcus agalactiae	CNCTC 10/84	W903_1422	MKTKKIIKKTKKKKSNLPFIILFLIGLSILLYPVVSRFYYTIESNNQTQDFERAAKKLSQ
Streptococcus agalactiae	NEM316 (serotype III)	gbs1475	MGGKFQKNLKKSVVLNRWMNVGLILLFLVGLLITSYPFISNWYYNIKANNQVTNFDNQTQ
Streptococcus agalactiae	NEM316 (serotype III)	gbs1476	MKTKNINKKTKKKKSNLPFIILFLIGLSILLYPVVSRFYYTIESNNQTQDFERAAKKLSQ
Streptococcus suis	05ZYH33	SSU05_2097	MMKRSQLRKGISKQSILLKLLFLFGLVVTLYPWISQYYYRYDSDYKIQSFYEQAQQLPSE
Streptococcus suis	05ZYH33	SSU05_2098	MIQKKSGKTTKKKKKSNIPFYLVFLIGFGILLYPHVSNFYYRYESDQLTNSFDQEKKTLA
Streptococcus suis	05ZYH33	SSU98_2100	MMKRSQLRKGISKQSILLKLLFLFGLVVTLYPWISQYYYRYDSDYKIQSFYEQAQQLPSE
Streptococcus suis	05ZYH33	SSU98_2101	MIQKKSGKTTKKKKKSNIPFYLVFLIGFGILLYPHVSNFYYRYESDQLTNSFDQEKKTLA
Streptococcus suis	BM407	SSUBM407_1951	MMKRSQLRKGISKQSILLKLLFLFGLVVTLYPWISQYYYRYDSDYKIQSFYEQAQQLPSE
Streptococcus suis	P1/7	SSU1882	MMKRSQLRKGISKQSILLKLLFLFGLVVTLYPWISQYYYRYDSDYKIQSFYEQAQQLPSE
Streptococcus suis	P1/7	SSU1883	MIQKKSGKTTKKKKKSNIPFYLVFLIGFGILLYPHVSNFYYRYESDQLTNSFDQEKKTLA
Streptococcus suis	SC84	SSUSC84_1903	MMKRSQLRKGISKQSILLKLLFLFGLVVTLYPWISQYYYRYDSDYKIQSFYEQAQQLPSE
Streptococcus suis	SC84	SSUSC84_1904	MIQKKSGKTTKKKKKSNIPFYLVFLIGFGILLYPHVSNFYYRYESDQLTNSFDQEKKTLA
Streptococcus suis	A7	SSUA7_1912	MMKRSQLRKGISKQSILLKLLFLFGLVVTLYPWISQYYYRYDSDYKIQSFYEQAQQLPSE
Streptococcus suis	A7	SSUA7_1913	MIQKKSGKTTKKKKKSNIPFYLVFLIGFGILLYPHVSNFYYRYESDQLTNSFDQEKKTLA
Streptococcus suis	GZ1	SSGZ1_1904	MMKRSQLRKGISKQSILLKLLFLFGLVVTLYPWISQYYYRYDSDYKIQSFYEQAQQLPSE
Lactobacillus casei	ATCC 334	LSEI_0457	MTKRTRRPLGLIDIVIGCLLLAGFGVLCYPFASDAYVSYQNQQVIDRYRQQEARKNQMVL
Streptococcus anginosus	SA1	DK43_00940	MKKFFNLLTNLTIIAGILIISYPVISQIYYNYQTKQEVKDFDSSAQKLTSEDVTERIKLA
Streptococcus equi subsp. zooepidemicus	H70	SZO_18280	MVKNNSAIKKQSFLTGFVFPLLFFLGLLIASYPLLSHYYYRVQQNRAVQAFETAKRQVDS
Streptococcus suis	SC070731	NJAUSS_1931	MIQKKSGKTTKKKKKSNIPFYLVFLIGFGILLYPHVSNFYYRYESDQLTNSFDQEKKTLA
Streptococcus suis	SC070731	NJAUSS_1930	MKLLFLFGLVVTLYPWISQYYYRYDSDYKIQSFYEQAQQLPSEEVLKRLEHARAYNQTLE
Streptococcus suis	S735	YYK_09080	MIQKKSGKTTKKKKKSNIPFYLVFLIGFGILLYPHVSNFYYRYESDQLTNSFDQEKKTLA
Streptococcus suis	S735	YYK_09075	MMKRSQLRKGISKQSILLKLLFLFGLVVTLYPWISQYYYRYDSDYKIQSFYEQAQQLPSE
Streptococcus suis	JS14	SSUJS14_2054	MIQKKSGKTTKKKKKSNIPFYLVFLIGFGILLYPHVSNFYYRYESDQLTNSFDQEKKTLA
Streptococcus suis	JS14	SSUJS14_2053	MMKRSQLRKGISKQSILLKLLFLFGLVVTLYPWISQYYYRYDSDYKIQSFYEQAQQLPSE
Lactobacillus casei	BL23	LCABL_05230	MTKRTRRPLGLIDIVIGCLLLAGFGVLCYPFASDAYVSYQNQQVIDRYRQQEARKNQMVL
Lactobacillus casei	Zhang	LCAZH_0487	MTKRTRRPLGLIDIVIGCLLLAGFGVLCYPFASDAYVSYQNQQVIDRYRQQEARKNQMVL
Lactobacillus casei	BD-II	LCBD_0521	MTKRTRRPLGLIDIVIGCLLLAGFGVLCYPFASDAYVSYQNQQVIDRYRQQEARKNQMVL
Lactobacillus casei	LC2W	LC2W_0523	MTKRTRRPLGLIDIVIGCLLLAGFGVLCYPFASDAYVSYQNQQVIDRYRQQEARKNQMVL
Lactobacillus casei	LOCK919	LOCK919_0549	MTKRTRRPLGLIDIVIGCLLLAGFGVLCYPFASDAYVSYQNQQVIDRYRQQEARKNQMVL
Lactobacillus casei	LOCK919	LOCK919_p16	MTKRTRRPLGLIDIVIGCLLLAGFGVLCYPFASDAYVSYQNQQVIDRYRQQEARKNQMVL
Lactobacillus casei	12A	LCA12A_2465	MTKRTRRPLGLIDIVIGCLLLAGFGVLCYPFASDAYVSYQNQQVIDRYRQQEARKNQMVL
Lactobacillus rhamnosus	GG	LGG_00441	MTKRTRRPLGLIDIVIGCLLLAGFGVLCYPFASDAYVSYQNQQVIDRYRQQEARKNQMVL
Lactobacillus rhamnosus	GG	LRHM_0425	MTKRTRRPLGLIDIVIGCLLLAGFGVLCYPFASDAYVSYQNQQVIDRYRQQEARKNQMVL
Lactobacillus paracasei subsp. paracasei	8700 2	LBPG_02641	MTKRTRRPLGLIDIVIGCLLLAGFGVLCYPFASDAYVSYQNQQVIDRYRQQEARKNQMVL
Lactobacillus paracasei	N1115	AF91_01535	MTKRTRRPLGLIDIVIGCLLLAGFGVLCYPFASDAYVSYQNQQVIDRYRQQEARKNQMVL

Clostridium novyi	-	NT01CX_0210	MIKKRISIVIIFIGILIFIYPKISELYYTNKQKRIIRKYDNLIENKFNTKTKQQIENKDI
Coprococcus catus	-	CC1_21250	MRKLIKLIGLFMVFFGIGVYLYPAVSDIYTKYRTNESIEEFYQKYQIDETESEKKSTDSE
Macrococcus caseolyticus	JCSC5402	MCCL_0366	MKPEKKTKKNRISNVIFGLIFFAGFSILIYPMISAVYYDYEASNEVNDFNKEVELLSDQQ
Streptococcus suis	GZ1	SSGZ1_1905	MIQKKSGKTTKKKKKSNIPFYLVFLIGFGILLYPHVSNFYYRYESDQLTNSFDQEKKTLA
Finegoldia magna	ATCC 29328	FMG_0189	MWKKIKHNFVFILIFLLGFGVMMYPVISRLYYRVDSANQVKEFDKAVKGLSKEELAKRLA
Finegoldia magna	ATCC 29328	FMG_0190	MKKSKLKENLKYVLIFLVGFAVAMYPLISRLYYRIEANSEVHDFDTNAKKMDKKEIDRRI
Eubacterium limosum	KIST612	ELI_4237	MIILVFLLGVLIMGYPFFSNWYNQILQNRLITEYNISSEALTPEDKDAEYNKCREYNQNL
Coriobacterium glomerans	PW2	Corgl_0469	MAKRRKGRLFASIIAALVFFVGVGCILYPYVSDWYQKYLQSKVIRNQQEIIQEKDRSSLE
Trueperella pyogenes	TP8	X956_01065	MGVVLVGILALIYPVVSTHYNNYKQREFALKYGNNVQNIDTSEREAALEAAREYNKRLPG
Trueperella pyogenes	TP6375	CQ11_06630	MNPDQTPAPTPRRSNRRATIAIGVVLLGILALIYPVVSTHYNNYKQREFALKYGNNVQNI

Panel B

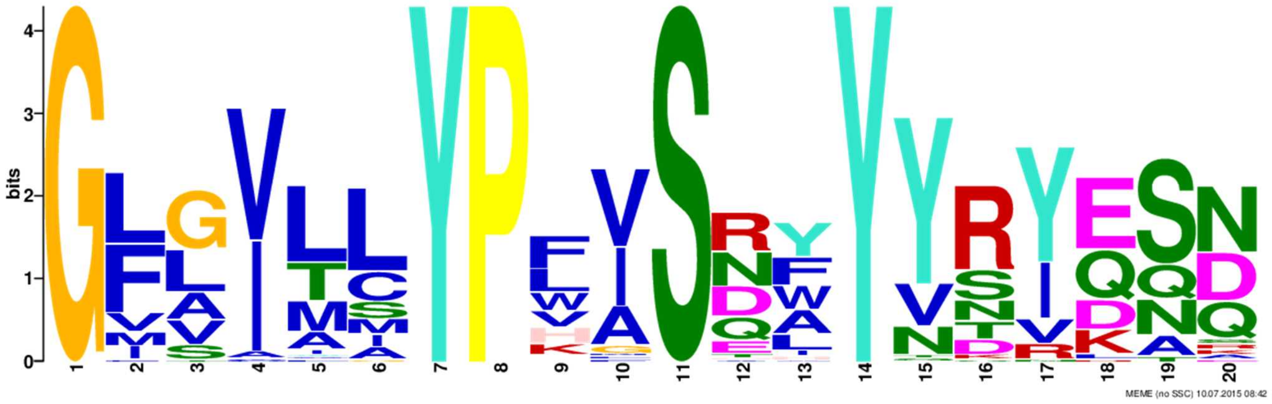

Supplement: S1 Fig — The input motif used for the search was the following one: G-x-x-x-x-x-Y-P-x-x-S-x-x-Y, which corresponds to the motif identified in L. rhamnosus GG SrtC1. We restricted our search to prokaryotic KEGG gene database and only considered proteins, where the motif start position was at most 30 amino acids from the start methionine. Out of 685 hits found in the prokaryotic KEGG gene database, 87 of them did have the GX5YPX2SX2Y motif located at the N-terminal domain. Interestingly, 69 out of 87 were proteins annotated as sortase enzymes. (A) The list below shows the complete list of proteins found through this search. (B) Using these 69 sequences, we also generated the LOGO motif using the MEME Suite. (PDF) [file pone.0153373.s001.pdf]
